# Update on Biomarkers for the Detection of Endometriosis

**DOI:** 10.1155/2015/130854

**Published:** 2015-07-09

**Authors:** Amelie Fassbender, Richard O. Burney, Dorien F. O, Thomas D'Hooghe, Linda Giudice

**Affiliations:** ^1^Department of Development and Regeneration, Organ Systems, KU Leuven, 3000 Leuven, Belgium; ^2^Department of Obstetrics and Gynaecology, Leuven University Fertility Centre, University Hospital Leuven, 3000 Leuven, Belgium; ^3^Departments of Obstetrics and Gynecology and Clinical Investigation, Madigan Healthcare System, Tacoma, WA 98431, USA; ^4^Division of Reproductive Biology, Institute of Primate Research, Karen, Nairobi 00 100, Kenya; ^5^Department of Obstetrics and Gynecology, University of California San Francisco, San Francisco, CA 94015, USA

## Abstract

Endometriosis is histologically characterized by the displacement of endometrial tissue to extrauterine locations including the pelvic peritoneum, ovaries, and bowel. An important cause of infertility and pelvic pain, the individual and global socioeconomic burden of endometriosis is significant. Laparoscopy remains the gold standard for the diagnosis of the condition. However, the invasive nature of surgery, coupled with the lack of a laboratory biomarker for the disease, results in a mean latency of 7–11 years from onset of symptoms to definitive diagnosis. Unfortunately, the delay in diagnosis may have significant consequences in terms of disease progression. The discovery of a sufficiently sensitive and specific biomarker for the nonsurgical detection of endometriosis promises earlier diagnosis and prevention of deleterious sequelae and represents a clear research priority. In this review, we describe and discuss the current status of biomarkers of endometriosis in plasma, urine, and endometrium.

## 1. Background

Endometriosis is a debilitating gynecologic disease characterized by the implantation of endometrial tissue in ectopic locations, including the pelvic peritoneum, ovaries, and bowel. The prevalence of endometriosis in reproductive age women is 2–10% [1] and as high as 35–50% in women with pain and/or unexplained infertility [2]. Endometriosis is a major cause of disability and significantly compromised quality of life in women and adolescents [3]. Symptoms include dysmenorrhea, dyspareunia (pain with intercourse), lower abdominal and/or back pain, dyschezia (pain with bowel movements), dysuria (pain with urination), and altered bowel habits [4]. Inflammation and innervation at sites of endometriotic lesions are implicated as causes of pelvic pain [5, 6]. Endometriosis is a major cause of infertility due to inflammation-associated reductions in oocyte quality and endometrial receptivity to embryonic implantation [7]. A heritable component to endometriosis is well supported, though the specific genes involved remain an area of active investigation. The risk for first degree relatives of women with severe endometriosis is six times higher than for relatives of unaffected women [8], and monozygotic twin studies demonstrate high concordance rates not only for histologically confirmed endometriosis but also for disease stage [9]. Though incomplete in accounting for the entirety of reported clinical manifestations of the disease, Sampson's theory of retrograde menstruation is the most widely accepted description of endometriosis pathogenesis [10]. This theory holds that endometriosis originates from the implantation of sloughed endometrial tissue refluxed into the pelvis via the fallopian tube(s) during menstruation.

Remarkably, the gold standard for the diagnosis of endometriosis remains direct visualization of lesions at surgery preferably coupled with histologic confirmation of endometrial glands and stroma in biopsies of suspected lesions, and this reality has significant consequences. A surgical diagnosis has multiple drawbacks, compared to a minimally invasive diagnostic, such as a blood or office-based test. These include risks inherent to the procedure (organ damage, hemorrhage, infection, and adhesion formation), as well as general anesthetic complications. Also patients need to travel to a hospital or outpatient surgicenter, with associated financial costs to the patient and the healthcare system, as well as prolonged time away from work and family. The requirement for invasive surgery for the diagnosis of peritoneal implants strongly contributes to an average latency of 7–11 years from onset of symptoms to definitive diagnosis [3, 11]. This delay in diagnosis is due, in part, to presumptive treatment of pain with oral contraceptives (OCPs) and nonsteroidal anti-inflammatory drugs (NSAIDs), as well as reluctance of physicians to refer women to gynecologists for definitive diagnosis, reluctance of women to confront their own pain for fear of a cancer diagnosis, and dismissal of pain, especially dysmenorrhea, as a “normal” event [12]. Delayed diagnosis and treatment has significant consequences, as endometriosis is more advanced in women whose diagnostic laparoscopy is delayed, supporting progression of disease over time [13]. Indeed, longitudinal placebo-controlled trials with second look laparoscopy have demonstrated that 71–83% of untreated lesions will progress or remain stable over a 12-month period [14]. At more advanced stages (stage III-IV of the revised American Fertility Society (rAFS) system) of endometriosis, the severity of pelvic pain may lead to hysterectomy often with oophorectomy. Endometriosis is the third leading cause of hysterectomy in the United States [15], and increasing evidence exists for the malignant transformation of ovarian endometriomas to ovarian cancer, particularly the clear cell and endometrioid subtypes [16].

In this review, we describe and discuss the current status of biomarkers of endometriosis in plasma, urine, and endometrium. This review aims to encourage optimized study design, data interpretation, and validation considerations in future biomarker development studies.

## 2. Diagnostic Test for Endometriosis

The gold standard for the diagnosis of peritoneal endometriosis has been visual inspection by laparoscopy followed by histological confirmation [7]. A noninvasive diagnostic test could be developed for serum or plasma, urine, endometrial, or menstrual fluid that can be recovered from the posterior vaginal fornix and from the cervix during speculum examination [17, 18]. A semi-invasive test could be developed in peritoneal fluid, obtained after transvaginal ultrasound guided aspiration or in endometrium obtained after transcervical endometrial biopsy [17, 18]. The most important goal of the test is that no women with endometriosis or other significant pelvic pathology are missed who might benefit from surgery for endometriosis-associated pain and/or infertility [17–19].

To achieve this, a test with a high sensitivity is needed, which is the probability of a test being positive when endometriosis is present. At present, such a test does not exist [17, 20].

A noninvasive test for endometriosis would be useful for women with pelvic pain and/or subfertility with normal ultrasound. This would include nearly all cases of minimal-mild endometriosis, some cases of moderate-severe endometriosis without clearly visible ovarian endometrioma, and cases with pelvic adhesions and/or other pelvic pathology, who might benefit from surgery to improve pelvic pain and/or subfertility [17–19].

Although there is consensus in the World Endometriosis Society that the development of a reliable noninvasive test is one of the top research priorities in endometriosis [11, 21], the development of such a test, from initial biomarker discovery to a clinically approved biomarker assay is a long, difficult, and uncertain process [22] which can be classified in four different phases as described below [17, 18].

*Phase I (Preclinical Discovery Phase)*. This phase consists of exploratory preclinical studies aiming to identify potential biomarkers. In endometriosis research, the state of the art in this field has recently been reviewed by May et al. [23]. 

*Phase II (Retrospective Validation)*. This phase consists of preclinical assay development and validation of a clinically useful noninvasive diagnostic test in the preclinical setting, as has been done in the context of endometriosis in a recent paper [24]. 

*Phase III (Prospective Clinical Validation and Determination of Clinical Utility)*. This phase establishes the diagnostic accuracy and predictive value in the target population, but this phase has not yet been reached in endometriosis biomarker research. 

*Phase IV (Commercialization)*. Product development by industry, which has not yet been done successfully for noninvasive endometriosis biomarkers.

Overall, most endometriosis biomarker studies have remained at the level of Phase I [23] and only a few have made it to Phase II studies. Clearly, there is a need for well-designed Phase II and Phase III trials to make progress in this field [18]. A clinically reliable test for endometriosis can be expected to have a profound impact on reduction of health care and individual costs by [18]

1. reducing time to diagnosis and the time wasted to see numerous health care professionals;
2. subsequently reducing the time before individualized specialist care is invoked;
3. subsequently reducing expensive hit-and-miss treatments;
4. subsequently reducing expensive fertility treatments if the disease is under control before fertility is impaired [25].

A worldwide study of costs caused by endometriosis indicates that the average annual total costs per woman are 9579 euros, of which 66% is caused by costs for productivity loss, while medication counts for only 10% of the total yearly costs [26]. Furthermore, endometriosis-associated symptoms generated 0.809 quality-adjusted life years per woman (the quality-adjusted life year is an outcome measure that accounts for the quantity and quality of life and that allows for comparison of outcomes between diseases), while the general population has 0.85–0.94 quality adjusted years per year [26–28]. In summary, a non- or semi-invasive test would not only reduce the cost associated with endometriosis but also improve the quality of life of women with endometriosis by allowing early diagnosis.

## 3. Blood Biomarkers 

Blood is an interesting potential source of biomarkers because it allows repeated measurements, is easily obtained, and is highly suitable for high-throughput measurements [29]. Putative endometriosis biomarkers are mostly glycoproteins, growth or adhesion factors, hormones, or proteins related to immunology or angiogenesis [17, 23, 30]. Despite extensive research, no single biomarker nor a panel of biomarkers in peripheral blood has been validated as a diagnostic test for endometriosis [17, 20].

Since the extensive review of May et al., 2010, research in endometriosis biomarkers has accomplished the successful validation of biomarkers in an independent sample dataset [24], but still no test for endometriosis is commercially available. As described earlier, different phases of biomarker discovery exist (Phase I–IV) and endometriosis biomarkers need to be validated prospectively in a clinical setting [17].

### 3.1. Glycoproteins

#### 3.1.1. Cancer Antigen- (CA-) 125

The use of CA-125 as blood biomarker for endometriosis has been examined extensively [23, 24, 30–36]. Several studies have demonstrated the utility of CA-125 for the diagnosis of endometriosis and its correlation to disease severity, especially endometriotic ovarian cysts [23, 32, 33]. However, CA-125 is not specific for endometriosis, being a tumor marker elevated in ovarian cancer [37, 38]. In addition to this lack of specificity, the sensitivity to detect all endometriosis stages is low [31]. According to a meta-analysis by Mol et al., the sensitivity for stage I–IV endometriosis was 50% and specificity was 72%. For stage III-IV endometriosis, a sensitivity of 60% could be obtained with a specificity of 80% [31].

CA-125 has been measured simultaneously with urocortin [34], chlamydia antibody [35], CD23 [39], and inflammatory cytokines [32]. However, none of these combinations provided a sufficiently high sensitivity or specificity for endometriosis and results remained unvalidated. The combination of CA-125, CA-19-9, and survivin mRNA showed promise, boasting a sensitivity of 87% and a 10% false positive rate [33]. A panel of CA-125, chemokine receptor (CCR) type-1 mRNA, and monocyte chemoattractant protein- (MCP) 1 showed a sensitivity of 92.2% and specificity of 81.6% to detect endometriosis [40]. CA-125 combined with interleukin-8 (IL-8) and tumor necrosis factors-*α* (TNF-*α*) in the secretory phase had a sensitivity of 89.7% and specificity of 71.1% in a study performed by Mihalyi and coworkers [36]. Ozhan et al. stated that a panel consisting of CA-125, syntaxin-5, and laminin-1 had 90% sensitivity, 70% specificity, and 88.7% accuracy to distinguish endometriosis patients (*n* = 60) from controls (*n* = 20) [41]. However, all these results remain to be validated.

Another ovarian tumor marker, CA-19-9, has been shown to be elevated in endometriosis and has a comparable or lower sensitivity than CA-125 for the detection of endometriosis [23]. A recent study showed a significant increase of CA-125 (*P* = 0.001), CA-19-9 (*P* = 0.015), and CA-15-3 (*P* = 0.017) in endometriosis cases (*n* = 50) versus controls (*n* = 35) [42]. ROC curve analysis showed that the area under the curve was the highest for CA-125 (0.938) [42]. For CA-19-9 a significant positive correlation with disease severity was found [42].

Recently, a panel of 4 biomarkers (CA-125, VEGF, Annexin V, and glycodelin/soluble intercellular adhesion molecule (sICAM)-1) showed a sensitivity of 74–94% and a specificity of 55–75% in a training set and after initial validation in an independent test set [24]. These results should be prospectively evaluated.

#### 3.1.2. Other Glycoprotein Markers

Follistatin, an inhibitor of activin, has been shown to be increased in endometriosis patients [23], especially in a subgroup of patients with ovarian endometrioma, and showed good sensitivity and specificity [43]. However, a follow-up study could not reproduce these results [44]. In a study conducted by Signorile and Baldi, Zinc (Zn)-alpha2-glycoprotein was identified by mass spectrometry (endometriosis cases *n* = 5, controls *n* = 5) as a possible biomarker for endometriosis and confirmed by ELISA to be differentially expressed (*P* = 0.019) in an additional cohort of endometriosis patients (*n* = 120) and healthy controls (*n* = 20) [45]. Reported sensitivity was 69.4% and specificity was 100% [45].

Glycodelin A, a promoter of neovascularization and cell proliferation, was examined in follicular phase serum of adolescent girls with endometriosis (*n* = 33) aged 13–19 alongside TNF-*α* and IL-6, but none of these proteins showed a different expression compared with adolescents without endometriosis (*n* = 17) [46]. A different study assessing glycodelin A found a significant increase in serum of women with endometrioma (*n* = 57) compared with control women undergoing sterilization or having benign ovarian cysts (*n* = 42) and demonstrated the potential use of glycodelin A as a biomarker for ovarian endometriosis with a sensitivity of 82.1% and a sensitivity of 78.4% [47]. Glycodelin was included in a biomarker panel proposed by Vodolazkaia et al., for the diagnosis of ultrasound-negative endometriosis [24], as mentioned in the section above on “Glycoproteins.”

### 3.2. Immunological Markers and Inflammatory Cytokines

Inflammatory and immunological markers have been implicated in the pathogenesis of endometriosis and have been examined extensively as possible biomarkers for endometriosis [23]. A plethora of cytokines has been assessed in the search for a noninvasive diagnosis of endometriosis, including IL-1, IL-6, IL-8, TNF-*α*, MCP-1, and interferon-*γ* (IFN-*γ*) [23]. In a study by Panoulis et al., no difference in the serum expression of the inflammatory markers CD40, CD40L and a disintegrin and metalloproteinase domain 8 (ADAM8) was detected between endometriosis patients (*n* = 47) and controls (*n* = 29) [48]. Values of T-helper pathway related interleukins IL-10, IL-12, IL-17, and IL-23 levels were comparable between infertile controls and endometriosis patients with infertility [49]. Contrasting results regarding changes in the complement system and soluble histocompatibility antigen (HLA) have been recorded [23]. The inflammatory marker C-reactive protein (CRP) has been shown to be upregulated [23], especially when examined with a high sensitivity assay making it possible to detect subclinical inflammation in women with endometriosis [50]. However, other studies could not find an upregulation [23, 51]. Significantly increased IL-4 serum values have been found in adolescents with endometriosis [52]. In a recent study, the inflammatory marker co-peptin was significantly (*P* = 0.002) higher in women with endometriosis (*n* = 50) than in women without endometriosis (*n* = 36) and was positively correlated with disease severity [42]. At a cut-off value of 251.18 pg/mL, its sensitivity to predict endometriosis was 65% and the specificity was 58.3% [42]. In a study by the same group, the inflammatory biomarker YKL-40 was significantly elevated (*P* < 0.001) in patients with endometriosis (*n* = 53) compared with patients without endometriosis (*n* = 35) and a positive correlation with disease severity was detected [53]. A study by Mihalyi et al. found a panel consisting of luteal plasma levels of IL-8, TNF-*α*, and CA-125 that was able to distinguish between 201 women with endometriosis and 93 controls with a normal pelvis with a sensitivity of 89.7% and a specificity of 71.1% [36]. In a study by Vodolazkaia et al., univariate analysis showed the differential expression of several cytokines and chemokines in 232 women with endometriosis and 121 controls [24]. However, no cytokines or chemokines were included in the final proposed panel of biomarkers after multivariate analysis [24]. Recently, the putative use of chemokines as biomarkers of endometriosis has been reviewed by Borrelli et al. [54]. In peripheral blood, IL-8, MCP-1, and RANTES showed potential as a biomarker, being significantly increased in endometriosis cases versus controls in, respectively, 46.1%, 50%, and 75% of the assessed studies [54]. However, no consensus exists on whether cytokines are suitable to discriminate endometriosis patients from patients with other pelvic pathology [24].

### 3.3. Oxidative Stress

Women with endometriosis might experience increased oxidative stress in the pelvic cavity due to the retrograde flow of menstrual erythrocytes that release iron upon rupture [55]. This has been confirmed by a number of groups that found alterations in a range of proteins related to oxidative stress. A significant reduction in serum has been reported for paroxonase (PON-I), high density lipoproteins [56], and plasma superoxide dismutase [57] and an increase of total cholesterol, triglycerides, low-density lipoprotein, lipid peroxidises [56], 25-hydroxycholesterol [58], heat shock protein 70b′ (HSP70b′) [59], and Vitamin E [57].

### 3.4. Cell Adhesion and Invasion

Levels of the sICAM-I have been suggested to rise during early stages of endometriosis (I-II) and decrease at stage III-IV [23]. Correspondingly, sICAM-I has been included in a panel with three other markers to diagnose endometriosis cases that could not be identified by preoperative ultrasound [24]. In addition, the cell adhesion molecule osteopontin was elevated in plasma for all disease stages [60, 61].

After initial cell adhesion, invasion of endometrial tissue fragments into the peritoneum may be facilitated through remodeling of the extracellular matrix by matrix metalloproteinases (MMPs) [62]. MMP-2 [63] and MMP-9 [64] have been found to be significantly increased in endometriosis patients versus controls. Moreover, advanced endometriosis is correlated with a higher MMP-2 expression [65]. In a study by de Sanctis et al., mRNA levels of MMP-3 were significantly higher in stage II–IV endometriosis cases than in controls [66]. The same study showed similar levels of MMP-9 and vascular endothelial growth factor A (VEGF-A) mRNA among cases and controls [66].

### 3.5. Angiogenesis

VEGF is an important regulator of angiogenesis. Its usefulness as biomarker for endometriosis is unclear, as some studies show elevated blood levels in endometriosis patients while other studies do not record a significant difference [23, 67]. Follow-up of patients with advanced endometriosis showed reduced VEGF-A levels after laparoscopic excision of the lesions [68, 69]. In another study, danazol treatment of endometriosis patients resulted in an increased VEGF concentration in plasma [70]. Despite these contrasting results, a recent study proposing a panel of biomarkers included VEGF in two panels to detect minimal/mild endometriosis with 80% sensitivity [24].

Pigment epithelium-derived factor (PEDF) is an inhibitor of angiogenesis and has neurotrophic and anti-inflammatory properties [71]. In a study by Chen et al., PEDF was significantly decreased in women with endometriosis (*n* = 43) compared with women without endometriosis (*n* = 28), independent of the phase of the cycle and correlated with pain symptoms [71]. Other growth factors, such as soluble epidermal growth factor (EGF) and platelet-derived growth factor (PDGF) have been investigated, but no difference was found between endometriosis patients and control women [23]. Hepatocyte growth factor (HGF) was suggested to be elevated in women with endometriosis [72], although this was not confirmed in an additional study [23, 73]. The serum concentration of its receptor, c-Met, was significantly higher in endometriosis patients (*n* = 130) than in controls (*n* = 39), in a stage dependent manner [74]. Elevations of fibroblast growth factor-2 (FGF-2), angiogenin, and soluble Flt-I (VEGFR-1) in serum of women with endometriosis have all been recorded [23].

### 3.6. Hormones

Contrasting evidence exists on prolactin, leptin, luteinizing hormone (LH), and adiponectin levels in endometriosis patients versus controls, showing either no difference, an increase (prolactin, leptin LH) or a decrease (adiponectin) [23, 75, 76]. No consensus exists on changes in steroid hormone levels [23].

### 3.7. Autoantibodies

Both total immunoglobulin levels and antiendometrial antibodies have been investigated as potential biomarkers for endometriosis of which the latter showed the most promising results, being more commonly present in endometriosis patients than in controls [23]. Likewise, specific antibodies against carbonic anhydrase, transferrin, *α*2-HS glycoprotein, lipid peroxide modified rabbit serum albumin, copper oxidized low-density lipoprotein and malondialdehyde-modified low-density lipoprotein, laminin-I, and cardiolipin have shown promise as potential endometriosis biomarkers [23]. Additionally, serum anti-PDIK1L [77] and anti-syntaxin 5 autoantibodies [78] were reported elevated in endometriosis patients. In patients with ovarian endometrioma, autoantibodies against Insulin-like growth factor 2 mRNA-binding protein 1 (IMP1) were significantly higher than in healthy controls [79]. In a study by Gajbhiye et al., 40 endometriosis patients were compared with 30 controls [80]. Autoantibodies against different epitopes of tropomyosin 3 (TPM3), stomatin-like protein 2 (SLP2), and tropomodulin 3 (TMOD3) were significantly elevated in the serum of endometriosis patients with both minimal/mild and moderate/severe disease [80].

### 3.8. miRNA

Micro RNAs (miRNAs) are highly conserved, short noncoding sequences that regulate gene expression at the posttranscriptional level. Generally, miRNAs repress transcription of their targeted messenger RNAs. With over 2200 distinct miRNAs identified to date, miRNA regulatory mechanisms are redundant, overlapping, and complex [81]. For example, most miRNA are able to regulate several hundred transcripts and several miRNA often regulate the same mRNA target [82]. Functional studies are increasingly clarifying the regulatory roles of individual miRNAs. The reduced proclivity of miRNA to degradation relative to mRNA [83] and strong correlation between tissue and serum miRNA expression evidenced in other disorders [84] are favorable features of miRNA in the context of biomarker potential.

Recently, miRNAs in peripheral blood have been suggested as potential endometriosis biomarkers, as reviewed by Fassbender et al. [85]. Reduced plasma levels of miR-17-5p, miR-20a, and miR-22 [86] and elevated plasma levels of miR-16, miR-191, and miR-195 [87] have been found in women with endometriosis compared with women without endometriosis. A study evaluating serum miRNA levels, found an elevation of miR-199a and miR-122 and a decrease of miR-145^*^, miR-141^*^, miR-542-3p, and miR-9^*^ in endometriosis patients compared with controls [88].

### 3.9. Proteomics

A variety of studies has been published regarding protein “fingerprints” for the diagnosis of endometriosis [23, 89–91]. A proteomic fingerprint model, based on three peptide peaks, had 91.4% sensitivity and 95% specificity to detect endometriosis when comparing 126 patients with endometriosis with 120 healthy controls [90]. Furthermore, this combination of peptide peaks was validated in an independent cohort, showing a sensitivity of 89.3% and a specificity of 90% [90]. A combination of 5 peptide peaks, discovered by surface-enhanced laser desorption/ionization time-of-flight (SELDI-TOF) mass spectrometry, detected endometriosis with a sensitivity of 88% and a specificity of 84% in the menstrual phase [91]. These studies have shown promising results; however, proteomics technologies are costly and time-consuming [23], and there is a need for better standardization and reproducibility of proteomic technologies before they can be used reliably in clinical research projects [17].

### 3.10. Metabolomics

Additionally, studies regarding the metabolome of endometriosis patients have been executed. Stearic acid was significantly reduced (*P* = 0.030) in endometriosis patients (*n* = 64) compared with controls (*n* = 74) [92]. In a study comprising patients with ovarian endometriosis (*n* = 40), eight metabolites and 81 metabolite ratios were significantly higher in the endometriosis group compared with healthy controls undergoing laparoscopy for sterilization (*n* = 52) [93]. The combination of hydroxysphingomyelin C16:1 and the ratio between phosphatidylcholine C36:2 to ether-phospholipid C34:2, adjusted for the effect of age and BMI, provided a sensitivity of 90.0% and a specificity of 84.3% for the detection of endometriosis [93]. A study comprising 22 women with minimal/mild endometriosis and 23 controls found higher values of Lactate, 3-Hydroxybutyrate, L-Alanine, Glycerophosphatidylcholine, L-Valine, L-Leucine, L-Threonine, 2-Hydroxybutyrate, L-Lysine, Succinic acid in the endometriosis group and lower values of Glucose, L-Isoleucine, and L-Arginine [94]. More research on the differences in the metabonomic profile between women with and without endometriosis should determine whether it could serve as a noninvasive diagnosis of endometriosis.

### 3.11. Circulating Cell-Free DNA

In a study by Zachariah et al., the concentration of circulating cell-free nuclear DNA was higher in endometriosis patients compared with the control group (*P* = 0.046), leading to the conclusion that circulating cell-free DNA may be a potential biomarker for endometriosis [95]. However, this assumption needs further investigation.

### 3.12. Cell Populations

A range of cell populations, including T cells, B cells, natural killer (NK) cells, macrophages/monocytes, and polymorphonuclear neutrophils, has been compared between endometriosis patients and healthy controls [23]. However, for none of these populations the utility as an endometriosis biomarker has been proven [23, 96]. Recently, a CD25^high^ forkhead box 3^+^ (FOXP3^+^) subset of CD4^+^ regulatory T cells has been shown to be decreased in peripheral blood of women with endometrioma (*n* = 17) compared with healthy controls (*n* = 15) [97]. Additionally, the potential use of circulating angiogenic cells as biomarkers for endometriosis has been examined, but no difference between endometriosis patients and controls could be detected [98].

## 4. Urine Biomarkers

For many diseases, urine has become among the most widely used clinical sample for biomarker discovery due to ease of access and less complex fluid composition. However, in endometriosis biomarker development, urine as an approach is significantly less targeted relative to blood. Since 2010, only 11% of reported endometriosis biomarker studies were urine-based [99].

Like serum, urine reflects an amalgam of systemic processes. Analysis of pooled urine from healthy men and women revealed that 70% of the urine proteins originate directly from the urinary system and the remaining 30% represent proteins from other organ systems filtered by the kidney [100]. Though legitimizing urine as a diagnostic medium, this finding also suggests potential for reduced specificity, and it will be important to assess the ability of a urinary assay to differentiate endometriosis from other inflammatory conditions.

Urine-based biomarker candidates measured by a variety of protein detection methods have been reported singularly or combined in a panel of markers. Creatinine-corrected soluble fms-like tyrosine kinase (sFlt-1) was found to be significantly elevated in the urine of women with endometriosis using enzyme-linked immunosorbent assays (ELISA) [101]. Using an immunoblot technique, Tokushige et al. demonstrated cytokeratin-19 (CK19) to be uniquely expressed in 11 urine samples from women with histologically proven endometriosis relative to samples from 6 women free of disease [102]. A larger prospective study was subsequently unable to confirm the diagnostic potential for urinary CK19 [103], possibly due to different specimen collection techniques or different subject characteristics. As in serum, matrix metalloproteinases (MMPs) have been investigated for association with endometriosis. A panel consisting of MMP-2, MMP-9, and MMP-9/neutrophil gealtinase-associated lipocalin was significantly elevated in a cohort of 33 women with endometriosis relative to expression in a group of 13 controls [104].

Relative to blood, urine evidences a significantly narrower dynamic range of proteins, thereby allowing more rapid preparation of specimens for proteomic interrogation. Additionally, the urine proteome is relatively stable for up to six hours at room temperature and for over 17 years stored at −70°C [105]. Using matrix assisted laser desorption/ionisation time-of-flight mass spectrometry (MALDI-TOF MS), several groups have reported differential peptide profiles in the urine of women with endometriosis relative to that of women without endometriosis at surgery [102, 106]. El-Kasti et al. identified a 3280.9 Da periovulatory peptide that differentiated all stages of endometriosis from controls with 82% senstitivity and 88% specificity. Tokushige et al. coupled MALDI-TOF with two-dimensional polyacrylamide gel electrophoresis (2D-PAGE) to reveal 12-fold higher expression of five proteins in affected women. Importantly, MALDI-TOF does not allow direct identification of peptides or proteins that are differentially synthesized or secreted, which is fundamental to further validation and clinical assay development, although protein pattern recognition holds promise for the future.

Advances in mass spectrometry (MS) technologies and bioinformatics have enabled protein analysis that can identify qualitative and quantitative differences in large numbers of lower abundance proteins. Cho et al. used 2D-PAGE and tandem MS to identify significantly higher levels of 22 urine proteins in women with endometriosis including vitamin D-binding protein, prealbumin, enolase-1, and alpha1-antitrypsin [107]. As individual analytes, these proteins evidenced insufficient sensitivity and specificity for use as a biomarker. Despite elevation in women with endometriosis, enolase-1 lacked sufficient diagnostic power as an individual analyte (sensitivity 56% and specificity 72%) in a separate study [108].

## 5. Endometrial Biomarkers

Though more invasive than serology, endometrial tissue is accessible via biopsy in the office setting and offers the potential advantage of improved specificity. Devices such as the Pipelle suction-based sampler are commonly used in the office without the need for anesthesia. The endometrium presents several unique characteristics with respect to biomarker discovery. First, the endometrium evidences remarkable sex steroid-driven cyclic variation and regenerative capacity. Whole genome profiling of normal endometrium revealed tremendous molecular variation between samples taken from the proliferative, early-secretory, midsecretory, and late-secretory phases of the menstrual cycle [109], and this basal cyclic variation in the endometrium must be accounted for in the interpretation of endometrial gene and protein expression signatures. An endometrial diagnostic assay is preferably obtained in the proliferative phase, as this avoids concerns regarding interruption of a nascent unanticipated pregnancy.

In addition to menstrual cycle phase, gynecologic conditions other than endometriosis have been shown to influence eutopic endometrial gene and protein expression. The endometrial transcriptome in women with endometriosis may have shared patterns of dysregulation with other inflammatory conditions such as hydrosalpinx [110] or other estrogen dependent diseases such as leiomyomata, endometrial polyps, or adenomyosis [111, 112]. Clustered pathologies may confound the interpretation of molecular measurements in the delineation of a biomarker unique to endometriosis. Consequently, screening and annotation of coexisting pathology is an important consideration in the biomarker discovery and validation process. A systematic review of over 200 potential endometrial biomarkers, including hormones and their receptors (*n* = 29), cytokines (*n* = 25), factors identified through proteomics (*n* = 8), and histology (*n* = 10) revealed sensitivity and specificity (reported in only 32 articles) ranging from 0 to 100% [113].

### 5.1. Endometrial Transcriptome

At the transcript level, significant differences in gene expression exist in eutopic endometrium from women with versus without endometriosis [114–117]. Both array-based global and targeted gene expression studies [113] have identified genes and pathways that may be involved in disease pathogenesis and reveal potential candidates for the development of an endometrial-based biomarker. Recently, whole genome microarray data involving 144 endometrial specimens from women with endometriosis or other benign gynecologic pathology (i.e., leiomyomata, endometrial polyp, and hydrosalpinx) and from women with surgically confirmed normal findings were used to develop menstrual cycle phase specific classifiers with high accuracy in the detection of both endometriosis and stage of disease [118]. In each cycle phase, specimens were partitioned into 80% construction and 20% independent validation sets for margin tree based training and testing of classifiers. Interestingly, relatively few genes were required to delineate endometriosis from other benign pelvic conditions and to classify disease severity. For example, the two best performing proliferative and early-secretory phase-specific disease classifiers achieved 100% accuracy using less than 100 genes for each disease classification decision. These highly informative gene sets provide a finite panel for biomarker development purposes. The delineation of endometriosis from other benign pelvic conditions represents an important strength of this study considering the high rate of clustering of estrogen dependent pathologies. Prospective validation in a large independent cohort of endometrial specimens collected at multiple centers is warranted.

### 5.2. MicroRNAs

MicroRNAs (miRNAs) evidence differential expression in the endometrium of women with versus without endometriosis and therefore offer potential as an endometriosis biomarker.

Like the endometrial transcriptome generally, microRNA expression in normal endometrium exhibits dynamic changes across the menstrual cycle. A comparison of miRNA array based profiles of human primary epithelial cells isolated from estrogen-dominant late proliferative (*n* = 4) and progesterone-dominant mid secretory (*n* = 4) phase endometrial specimens identified 24 differentially expressed miRNAs [119]. This finding highlights both the prospect for miRNA dysregulation in the pathogenesis of endometrial disorders and the importance of accounting for menstrual cycle phase in the interpretation of miRNA profiles in biomarker discovery protocols.

Global differential expression of miRNAs in eutopic compared with ectopic endometrium has been evaluated by several groups [120–124]. Pan et al. identified differential expression of 48 miRNAs in a microarray analysis of early to mid-secretory eutopic endometrial tissues from endometriosis-free volunteers (*n* = 4), and from eutopic (*n* = 4) and ectopic (*n* = 8) endometrial tissues from women with endometriosis. Using arrays probing 377 miRNAs to compare eutopic and ectopic (peritoneal) endometrium from seven women with stage II–IV endometriosis, Teague et al., detected dysregulation of 22 miRNAs, with predicted cognate mRNA targets known to be involved in endometriosis pathogenesis [121]. Interestingly, the dysregulation of miRNAs was cycle phase independent, though the relatively small sample size limited definitive correlation. Similar to peritoneal disease, ovarian endometriosis evidenced differential expression of miRNAs relative to paired eutopic endometrium in several studies [122, 123]. A more recent study revealed 156 miRNAs differentially expressed between endometriotic tissue and normal endometrium, including twelve miRNAs known to be involved in fibrinolysis and angiogenesis [124]. These studies highlight molecular pathways that may be associated with the development of endometriosis as well as the changes in expression signature that exist in ectopically located endometrial tissue.

In contrast to studies comparing miRNA expression in eutopic versus ectopic endometrium, relatively few studies have compared miRNA expression in eutopic endometrium from women with and without surgically confirmed endometriosis [120, 125, 126]. In a parallel miRNA-mRNA array based comparison of three control early secretory phase endometrium (*n* = 3) with endometria from four women with moderate-severe endometriosis, six downregulated endometriosis associated miRNA were identified from the miR-9 and miR-34 miRNA families [125]. MiR-9 is also dysregulated in endometrioid ovarian cancer, with which endometriosis is associated. Though strengthened by the stringency of including only surgically documented presence or absence of advanced stage endometriosis, the study is limited by inclusion of control endometrium from women with coexisting intramural leiomyomata which could confound delineation of endometriosis-specific miRNA differences. This group further compared miRNA expression in women with mild and severe endometriosis and found increased endometrial expression of miR-21 and DICER in the more advanced stage of the disease [126]. In addition to these global miRNA studies, others have compared endometrial expression of individual miRNAs in the eutopic endometrium of women with and without endometriosis. In general, individual miRNAs are selected on the basis of biological plausibility in the pathogenesis of the disorder. For example, miR-135a (proliferative phase) and miR-135b (proliferative and secretory phases) were investigated due to their predicted interaction with Homeobox protein (HOX) A10 [127]. The overexpression of these miRNAs correlated with the downregulation of HOXA10 in endometrium from women with endometriosis. Direct regulation of HOXA10 by miR-135a/b was subsequently confirmed by luciferase assay in cultured endometrial stromal cells. Other miRNAs and predicted cognate mRNAs demonstrating differential expression in eutopic endometrium from women with and without endometriosis include miR-23a/CYP19A1 and miR-542-3p/COX2 [128], miR-126/CRK [129] and miR23a/NR5A1 [130]. Notably, the reports of miR-23a expression in eutopic endometrium from women with and without endometriosis showed opposite directions of dysregulation, with one study involving proliferative endometrial specimens and the other including only early to midsecretory samples.

The demonstration of aberrant microRNA expression profiles in the eutopic endometrium from women with endometriosis may yield promising biomarker targets. However, independent validation and replication of miRNA dysregulation in phase specific comparisons are needed. To date, the utility of miRNAs as biomarkers for endometriosis has not been specifically tested.

### 5.3. Endometrial Proteome

Several groups have reported unique proteomic profiles using the SELDI-TOF MS platform in eutopic endometrial specimens from women with and without endometriosis [131, 132]. Importantly, the SELDI-TOF MS methodology provides differential proteomic profiles in the form of mass/charge (*m*/*z*) peaks without attendant characterization of the peptides or proteins. In 2006, the first of these reports described reduced expression of a protein peak in secretory phase endometrium from women with mild endometriosis relative to controls [133]. A larger study identified differential expression of 32 peptide peaks in secretory phase endometrium from 10 women with endometriosis (all rAFS stages) compared to that of 6 healthy women [134]. Wang et al. performed proteomic profiling of endometrium from 13 women with and 13 women without endometriosis identifying five differentially expressed peptide peaks (5.385* m*/*z*, 5.425* m*/*z*, 5.891* m*/*z*, 6.448* m*/*z*, and 6.898* m*/*z*) that collectively showed 91.7% sensitivity and 90% specificity in the diagnosis of endometriosis [132]. In the largest study to date involving a total of 53 endometrial samples, a panel of three differentially expressed peptide peaks (16.069* m*/*z*, 15.334* m*/*z*, and 15.128* m*/*z*) diagnosed endometriosis (all rAFS stages) with 87.5% sensitivity and 86.2% specificity [135]. Another study characterized a panel of five differentially expressed peptide peaks in secretory phase endometrium (1.949* m*/*z*, 5.183* m*/*z*, 8.650* m*/*z*, 8.659* m*/*z,* and 13.910* m*/*z*) to have 89.5% sensitivity and 90% specificity for the diagnosis of any stage endometriosis [136]. In a unique concomitant assessment of the endometrial transcriptome and proteome, Fassbender et al. described a panel of differentially expressed peptide peaks (2072* m*/*z*, 2973* m*/*z*, 3623* m*/*z*, 3680* m*/*z,* and 21133* m*/*z*) in the early secretory endometrial proteome of women with versus without endometriosis as diagnostic of endometriosis (all rAFS stages) with 91% sensitivity and 80% specificity [131]. Though the differentially expressed* m*/*z* peaks identified among separate groups showed no overlap, important methodological differences are apparent. Specifically, menstrual cycle phase of endometrial samples was not specified in several of the studies [132, 135], and only one randomly divided endometrial samples into training and test sets [131]. To date, none of the differentially expressed peptide peaks have been validated in an independent study cohort to which investigators are blinded as to patients' disease status. Of paramount importance toward the development of a clinical laboratory protein assay such as ELISA is the identification of differentially expressed peptides and proteins.

### 5.4. Neuronal Marker

Clarification of the role of neuroangiogenesis in endometriosis has led to investigation of the biomarker potential for nerve fibers in eutopic endometrial samples. Nerve fibers were first detected in peritoneal endometriotic lesions and these were thought to contribute to associated dysmenorrhea [137]. Immunohistochemical detection of the protein gene product 9.5 (PGP9.5), a highly specific pan-neuronal marker, was described at peritoneal lesions developing from surgically transplanted uterine horn segments in a rat model of endometriosis [138]. These findings in endometriotic lesions led to assessment for differences in nerve fiber density in the eutopic endometrial microenvironment. In a study of sharp curettage and full thickness hysterectomy specimens, PGP9.5 immunostained nerve fibers were detected in the functional endometrial layer from all women with surgically confirmed endometriosis but none of the specimens from unaffected controls, and this finding was cycle phase independent [139]. These striking findings were followed by two independent studies assessing the detection of endometrial nerve fibers as a diagnostic test for endometriosis [140, 141]. In a study of archived biospecimens, the density of nerve fibers was fourteen times higher in the endometrium of women with rAFS stage I-II endometriosis relative to that of healthy women, and the combination of PGP9.5, substance P, and vasoactive intestinal peptide was 95% sensitive and 100% specific for the diagnosis of endometriosis [141]. In a double blind study of endometrial pipelle samples from 99 consecutive women undergoing laparoscopy for pelvic pain and/or infertility, immunohistochemical detection of PGP9.5 demonstrated 98% sensitivity and 83% specificity for the finding of endometriosis at surgery [140]. Importantly, nerve fibers were not observed in other benign gynecologic conditions to include endometritis, leiomyomata, or endometrial polyps. Meticulous sampling technique for collection and proper orientation of the functional endometrial layer were methodologic points of emphasis. These results were confirmed in a study of 27 prospectively collected eutopic endometrial specimens using identical sampling and detection methods [142]. However, the adoption of this method in a clinical laboratory failed to recapitulate the accuracy of endometrial PGP9.5 immunohistochemistry in the diagnosis of women with endometriosis [143]. Functional endometrial layer nerve fibers assessed by PGP9.5 immunostaining were detected in 9 of 45 (22%) of histologically confirmed cases of endometriosis and in 6 of 21 (29%) of women without endometriosis. Potential explanations for the discrepant results include curette rather than pipelle-based sampling, inability to orient the curette fragments for functional layer assessment and inclusion of women undergoing hormonal treatment. Nonetheless, the detection of nerve fibers in 29% of women without endometriosis raised concerns regarding the assay's specificity [143]. The specificity of endometrial nerve fiber density was further challenged by the finding of similar endometrial innervation and neuronal growth fibers in women with adenomyosis, with expression reported to be more correlated with pelvic pain than diagnosis [144]. Studies involving larger populations are needed to validate the utility of endometrial nerve fiber density as a biomarker for endometriosis.

## 6. Standard Operating Procedures

Many centres worldwide have been collecting blood or other body fluids such as peritoneal fluid, endometrial fluid, and menstrual fluid, as well as tissue samples—in particular ectopic and eutopic endometrium—from women with and without endometriosis, for a variety of research purposes [145–147]. The adoption of validated, internationally agreed upon standard operating procedures (SOPs) for tissue sample collection, processing and storage, and standardized phenotypic and other patient data collection, are crucial to optimise sample quality, reduce variability, and enable cross-centre studies [17, 113, 148]. This can allow researchers to overcome the main pitfalls in the study design and methodology such as small sample size, lack of relevant clinical information inconsistency in sample handling and storage, and technical control of preanalytical sample variability, which contribute to controversial study results in endometriosis research [17].

Recently, the World Endometriosis Research Foundation (WERF) Endometriosis Phenome and Biobanking Harmonisation Project (EPHect) has developed a consensus on standardisation and harmonisation of phenotypic surgical/clinical data and biologic sample collection methods in endometriosis research [146, 147, 149, 150]. This consensus [146, 147, 149, 150] was developed on the basis of publicly available SOPs from general large-scale biobanking efforts, on a systematic literature search in PubMed and Google search, on published SOPs for endometriosis related biobanking [17, 151], and on personal biobank experience from study participants. Two types of SOPs were developed: standard recommended and minimum required. “Standard” collection SOPs should be adopted where possible, as they will yield results that are least prone to variation and degradation of the samples; “minimum” SOPs should be used by all individuals starting an endometriosis biobank as they provide the fundamentals for standardization required as an absolute minimum requirement given unavoidable logistical and budgetary circumstances. All questionnaires and SOPs produced by the WERF EPHect Working Group are freely available for use by investigators on the WERF EPHect website: http://endometriosisfoundation.org/ephect/ [146, 147, 149, 150].

## 7. Conclusion

Despite the plethora of studies on endometriosis biomarkers, neither a single biomarker nor a panel of biomarkers has been validated for a noninvasive diagnostic test with sufficient sensitivity and specificity [17]. A first step toward validation of biomarkers has been made [24, 140]; however, further studies are needed to develop a clinically useful test. Currently, biomarker research in endometriosis is still lacking reproducible data with high sensitivity and specificity. In addition, limitations derive from small sample size and suboptimal characterisation of specimens (no breakdown according to menstrual phase or lesion phenotype).

Discovery of new biomarkers and validation of putative biomarkers are crucial to make progress in the field [17] and are top research priorities for endometriosis proposed in 2009 and 2013 by highly ranked researchers [11, 21].
